# Chlorpyrifos Occurrence and Toxicological Risk Assessment: A Review

**DOI:** 10.3390/ijerph191912209

**Published:** 2022-09-26

**Authors:** Elżbieta Wołejko, Bożena Łozowicka, Agata Jabłońska-Trypuć, Marta Pietruszyńska, Urszula Wydro

**Affiliations:** 1 Department of Chemistry, Biology and Biotechnology, Bialystok University of Technology, Wiejska 45A Street, 15-351 Białystok, Poland; 2 Institute of Plant Protection—National Research Institute, Chełmońskiego 22 Street, 15-195 Białystok, Poland; 3 Department of Ophthalmology, Medical University of Białystok, M. Skłodowskiej-Curie 24A Street, 15-276 Białystok, Poland

**Keywords:** CPF, toxicity, risk assessment, environment

## Abstract

Chlorpyrifos (CPF) was the most frequently used pesticide in food production in the European Union (EU) until 2020. Unfortunately, this compound is still being applied in other parts of the world. National monitoring of pesticides conducted in various countries indicates the presence of CPF in soil, food, and water, which may have toxic effects on consumers, farmers, and animal health. In addition, CPF may influence changes in the population of fungi, bacteria, and actinomycete in soil and can inhibit nitrogen mineralization. The mechanisms of CPF activity are based on the inhibition of acetylcholinesterase (AChE) activity. This compound also exhibits reproductive toxicity, neurotoxicity, and genotoxicity. The problem seems to be the discrepancy between the actual observations and the final conclusions drawn for the substance’s approval in reports presenting the toxic impact of CPF on human health. Therefore, this influence is still a current and important issue that requires continuous monitoring despite its withdrawal from the market in the EU. This review traces the scientific reports describing the effects of CPF resulting in changes occurring in both the environment and at the cellular and tissue level in humans and animals. It also provides an insight into the hazards and risks to human health in food consumer products in which CPF has been detected.

## 1. Introduction

Chlorpyrifos (CPF) belongs to a group of organophosphorus pesticides, which exhibit a wide spectrum of biological activity [1]. This compound is applied in various agricultural and horticultural crops and in households to combat biting and stinging-sucking pests. They act on pests by contact, ingestion, and inhalation, and on the plant surface and inside [2]. Upon entering organisms, CPF inhibits the activity of acetylcholinesterase (AChE), one of the most important enzymes influencing the peripheral and central nervous system in pests. It may also affect the human nervous system in a similar way, contributing to the alteration of normal biochemical and physiological conditions [1].

The application of CPF for agricultural purposes results in its dispersion into various environmental components such as air, soil, surface water (rivers, canals and lakes), and groundwater, disrupting ecosystem functioning [3]. Due to its lipophilicity, CPF has the ability to penetrate the epidermis and enter the animal’s body, entering tissues, organs, milk, and other body liquids. In addition, CPF residues have also been detected in fruit, grains, and vegetables. The presence of CPF residues in soils, natural waters, food, and animals and humans is not a new topic, but studies related to the occurrence and biodegradation of this pesticide in different environmental elements and its impact on the ecosystem are the object of research of many researchers even today [4,5].

Interestingly, on a nearly daily basis, humans are exposed to CPF because of the common use of this compound in households, for example, to control pests such as flies, cockroaches, and ants [6]. Long-term human exposure may result in various health abnormalities, mainly in the nervous, cardiovascular, and respiratory systems [7]. The residues of CPF and its metabolites can be found in human tissues and urine samples from both people employed in agriculture and those who are not in contact with it professionally [8,9].

WHO warns that every year, as many as 2.5 million people worldwide suffer from acute poisoning with pesticides and 0.2 million people die. Among all residues of pesticides detected in the environment, organophosphorus pesticides constitute over 50%. Three groups of people are mainly exposed to CPF: workers producing these chemical preparations, farmers [2,10], and unaware consumers who consume food with pesticide residues. On the basis of various studies, the European Commission (EC) verifies the authorizations or trade permits for pesticides, which are subsequently approved or withdrawn from use. Moreover, based on the research obtained for CPF, on January 16, 2020, the European Commission Regulations No 2020/18 and 2020/17 [11] came into force, according to which CPF were withdrawn. Insecticides containing this active substance were on the market until April 1, 2020, while the deadline for their use was April 16, 2020. After this date, these insecticides cannot be placed on the market or used in the EU, and they can only be utilized by specialized and licensed companies [10]. However, there are reports that CPF is still used on insects in India for desert locus control of crops, acacia, and other trees in concentrations of 240 g/ha. Despite the fact that the use of CPF on crops will be banned in Egypt at the end of 2022, it will still be possible to be use it on cotton and against locusts and termites [12]. In addition, Health Canada is phasing out the sale of products containing CPF by 2022 and allows the use of CPF products until 10 December 2023 (Health Canada Pest Management Regulatory Agency 2020). Whereas, in Australia, assessments are being made related to the toxicology, safety, residues, and environmental impact, which should be published in mid-2022 [12]. Moreover, this pesticide is banned for use on crops in Vietnam, Morocco, Sri Lanka, Saudi Arabia, Indonesia, Palestine, Switzerland, Thailand, and Turkey [13].

The toxicity of CPF is still the subject of toxicological research, and so far, a significant amount of information has been gathered on its main mechanisms of action and various short-term side effects in both humans and animals. Therefore, more scholarly attention should be paid to the detection of long-term health effects associated with exposure to low levels of CPF. In this review, an analysis based on the current literature is conducted to shed new light on the long-term health effects of exposure to CPF. This problem is discussed on the basis of research studies on suitable animal and cellular models, which not only provides highly relevant information on CPF-induced toxicity but also on the developmental effects and molecular mechanisms of action.

## 2. Materials and Methods

This publication uses selected reports of organizations such as the United States Environmental Protection Agency (US EPA), the European Food Safety Authority (EFSA), and European Commission (EC), which provide guidance relevant for the regulation of human risk assessment. In addition, risk profile data was analyzed for chlorpyrifos. This data was extracted from PubMed to May 2021 using the following keywords: “chlorpyrifos”, “risks for human health”, “toxic effects of chlorpyrifos”, and “chlorpyrifos in food and environment”. In the last 20 years, scholars have contributed a total of over 2034 studies on the toxic effects of CPF and the present manuscript is based on 175 of them.

## 3. Results and Discussion

### 3.1. The Behavior of Chlorpyrifos in the Environment

The application of CPF on plants involves about 1% of the product reaching the target pest while the remainder comes into contact with soil, where it is transformed and metabolites are formed [4]. According to Jaiswal et al. [14] and Huang et al. [15], CPF is strongly absorbed by soil and is poorly leached from soils due to its difficult solubility in water. Volatilization from the soil surface also contributes to the loss of chlorpyrifos. As reported by Singh et al. [16], the half-life of chlorpyrifos in soil ranges from 20 to 120 days, with the formation of 3,5,6-trichloro-2-pyridinol (3,5,6-TCP) as the main degradation product. Other data indicate that the half-life can range from 2 weeks to more than 1 year [17]. This high interchangeability of the half-life is related to the soil properties, which include the soil type, pH, moisture, temperature, organic matter and organic carbon content, and the microbial metabolism of CPF. The degradation of CPF is increased by higher soil temperatures with lower organic matter contents and lower acidity. Another important factor is the characteristics of the chlorpyrifos-based plant protection product (e.g., its composition, surfactant content, and other auxiliary compounds) and its method of application [14,18,19].

Literature data indicate that CPF can affect the soil microflora population and inhibit the cycling of important soil nutrients, including nitrogen fixation by bacteria [20,21]. The production of reactive oxygen species (ROS) induced by CPF directly contributes to the reduction in nitrase activity by inhibiting the expression of genes related to soil nitrogen fixation [4]. Riah et al. [22] reported that CPF can also affect the activity of soil enzymes such as phosphatases and β-glucosidase. In addition, the metabolites generated by CPF degradation may exhibit greater toxicity than the original compound. Chlorpyrifos-oxon (CPF-ox) and 3,5,6-TCP have high toxicity to microbial populations and soil enzymatic activity [23]. A study by Guo et al. [24] showed that the presence of CPF in the rhizosphere can affect the mobility of antibiotic resistance genes. Moreover, recent studies indicate that CPF has the ability to form bound residues (BRs) in soils, which may be due to physical entrapment of the parent compound and its main metabolite 3,5,6-TCP, captured mainly by humic acids. However, for the time being, the stability and activity of BRs requires additional research [25].

One method of CPF degradation in soil is bioremediation with microorganisms such as *Pseudomonas* sp.*, Enterobacter, Serratia, Alcaligenes, Sphingobacterium, Gordonia, Paracoccus*, and *Mesorhizobium*. The main breakdown products of CPF include 3,5,6-TCP and diethyl thiophosphoric acid (DETP) [26]. As endophytes, some of the listed microorganisms further promote plant growth and nullify the phytotoxicity of pesticides [27]. Ahmad et al. [28] reported the beneficial effects of bioremediation with *Bacillus pumilus* C2A1 with ryegrass phytoremediation against CPF in soil.

Sanchez-Hernandez et al. [23], on the other hand, studied the effect of *L. terrestris* in CPF-contaminated soil. They found that *L. terrestris* did not significantly affect CPF degradation but had a beneficial effect on soil enzyme activity. 

Recent studies indicate the use of nanomaterials to remove CPF from soils. Nowadays, nanomaterials in the form of nanotubes or nanowires have received significant interest in studies on the remediation of various pesticides (including CPF) and their real-time applications [29].

**Figure 1:**
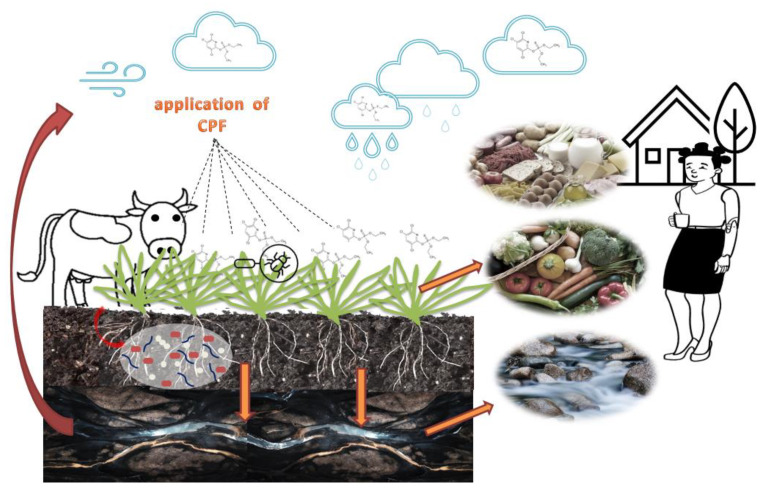
The occurrence and permeation of CPF in the environment.

Agrochemicals applied to soil can either leach through the soil profile and consequently enter groundwater or enter surface water, where they can affect groundwater (Figure 1). The main cause of pesticides entering the aquatic environment is rainfall, which causes leaching from areas where the pesticide has been applied [30]. In the case of CPF, it is characterized by its hydrophobicity and ability to bind with the organic fractions of environmental matrices. However, despite these properties, CPF has been detected in natural waters in different parts of the world [31,32]. CPF is stable in weakly acidic and neutral solutions, but it is hydrolyzed by strong bases [33]. Its presence in waters depends on the land topography, rainfall, agricultural practices, and its properties, among other factors [34]. CPF has been detected in surface water, seawater, and rainfall. Zhong et al. [35] analyzed arctic air and seawater during an oceanographic cruise from the North Pacific to the Arctic Ocean. They confirmed the occurrence of CPF in this part of the world at concentrations ranging from 0.08 to 0.85 pg/L in seawater and 0.5 to 2 pg/m^3^ in air. In general, lower concentrations were reported in marine waters than in surface waters. However, relatively high concentrations of CPF were detected in rainwater and ranged from 30 to 200 ng/L [15].

According to Kumar et al. [36], agricultural land used for vegetable and cotton cultivation plays a key role in CPF water pollution. A study by Hossain et al. [34] showed that the CPF content in lake waters from agricultural lands of Bangladesh ranged from 3.27 to 9.31 μg/L. The half-life of CPF in water varies and is approximately 0.5 days [37]. As reported by Lockridge et al. [38], the pH-dependent half-life of CPF-ox was 20.9 (pH 8) and 6.7 days (pH 9). The presence of CPF in the aquatic environment has a number of consequences, including ecotoxicological effects (genotoxicity, neurotoxicity, oxidative stress) on freshwater organisms [15].

The degradation of CPF in water, as in soil, can occur through a biodegradation process involving microorganisms such as *Pseudomonas, Burkholderia*, and *Brevundimonas* [34]. Research is also being conducted on the removal of CPF from water using microplasma [39]. In addition, Romeh [40] conducted research on the removal of CPF and 3,5,6-TCP from water using iron nanoparticles derived from *Ficus sycomorus* deposited on adsorbents of naturalized origins (e.g., wheat bran, rice straw ash) in combination with phytoremediation using *Plantago major*.

Literature data indicate that CPF can also be transferred freely with air currents. CPF in the air reacts with •OH radicals by photolysis and is converted into CPFox, with a half-life of 12 h. There are also reports that CPF can also be transformed into CPF-ox by reaction with ozone and nitrate radicals. The longer duration of CPF-ox in the air compared to CPF (3 h) may be another negative factor of its impact on the body of animals and humans. The distance that CPF or CPF-ox can cover in the air largely depends on the meteorological conditions such as wind speed, UV radiation, and air temperature [3,13]. Mackay et al. [3] indicated that CPF was detected in air samples both in regions where it was applied to crops and at locations away from the application areas (Figure 1). This may suggest that CPF migrates easily with air currents. Furthermore, research conducted in 2019 in Norway found that the compound was detected in house dust samples at levels from <3.0 to 1300 µg/kg dw [12]. This is of major concern because with air currents and then through rainfall, CPF may be detected in soil, water, and plants, further endangering human health. 

Plants are a very important part of the environment because they provide humans with valuable nutrients. Therefore, pesticide residues, including CPF, on plants can significantly affect human health [41]. Literature data indicate that studies are being conducted on the occurrence of CPF and its fate in plants. In addition to the negative effects of CPF on plants, there are also reports of its positive effects. At lower concentrations, it has been found to increase all growth parameters in seedlings [42]. A research study by Parween et al. [43] indicated that a lower dose of CPF was found to stimulate root and shoot length, and increase the nitrate reductase (NR) activity, nitrate content, and sugar-soluble protein while at higher concentrations, CPF had a significantly negative effect on all of the above parameters.

After foliar application, CPF metabolism in plants occurs through hydrolysis of P-O-pyridinol to 3,5,6-TCP. Literature data indicate that CPF in plants metabolizes rapidly and the first products are formed within 24–48 h after its application [44]. Some researchers have found that the part of CPF that is metabolized to 3,5,6-TCP may remain in the plant in the form of glycoside conjugates. However, other scholars have indicated that CPF-ox is immediately formed after CPF’s penetration through the roots. Furthermore, in the roots, CPF can be hydrolyzed to 3,5,6-TCP and DETP by cleavage of the phosphorus bond [45]. CPF-ox and 3,5,6-TCP are the two main CPF degradation metabolites identified in plant leaves and roots [44].

### 3.2. Degradation Pathways of CPF

**Figure 2:**
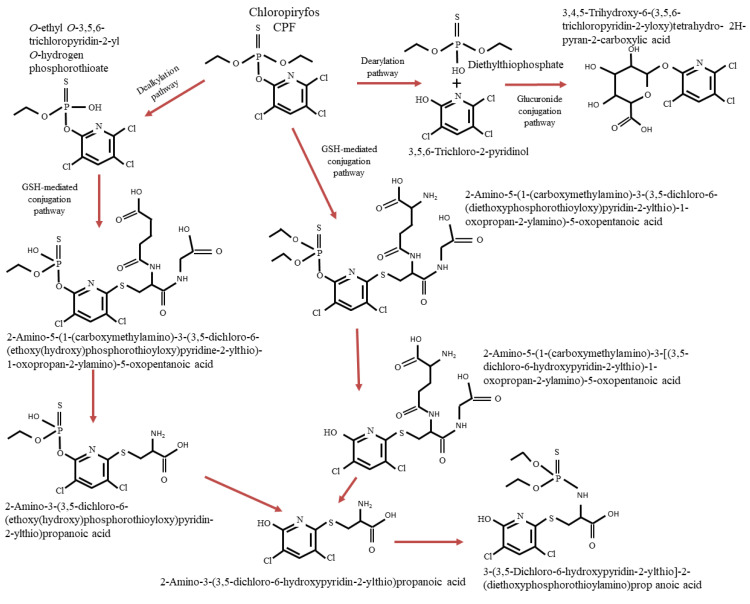
Biotransformation pathways of CPF in hepatocytes [49].

Studies carried out according to the ADME protocol (absorption, distribution, metabolism, excretion) indicate that over 70% of orally administered CPF is absorbed while dermally administered CPF was well absorbed in mice (>60%) but poorly absorbed in humans (<3%) [46,47]. After absorption, CPF is rapidly metabolized and excreted (27 h half-life in humans) mainly in the urine as 3,5,6-TCP and conjugates that hydrolyze to 3,5,6-TCP. Studies on radiolabeled material indicate that chlorpyrifos and its metabolites do not selectively accumulate in any tissue. Prior exposure had no effect on the distribution of CPF and no significant dose or gender differences were observed in its metabolism [48]. In hepatocytes, this substance is biotransformed by different pathways such as dearylation, dealkylation, glucuronidation, and GSH-dependent conjugation [49] (Figure 2). The mechanism of CPF toxicity is related to its ability to irreversibly inhibit AChE activity in tissues [2], accumulating acetylcholine, an excess of which causes continuous triggering of the acetylcholine receptor, affecting the functioning of the somatic and autonomic nervous systems [50]. However, Shenouda et al. [51] showed that inhibition of AChE does not explain all the symptoms of CPF intoxication because exposure to CPF can affect other systems, such as the hematological, immune, and reproductive systems [52]. 

Desulfuration of CPF to CPF-ox, which is dependent on the cytochrome P-450 molecule, occurs in the animal body at the initial stage of biotransformation pathways because active sulfur atoms irreversibly bind with cytochrome P-450, catalyzing the reaction. The created CPF-ox is regarded as the principal and most toxic metabolite of CPF, which is responsible for cholinesterase inhibition. Next, hydrolysis is followed by inactivation of oxon to form 3,5,6-TCP, diethyl phosphate (DEP), and DETP [49,51]. As reported by Smith et al. [53], 3,5,6-TCP, which emerges during the degradation of CPF, is considered a detoxification pathway. Eleršek and Filipič [54] stated that the CPF-ox and 3,5,6-TCP ratio in the body during CPF degradation is species-related and depends on the age, sex, P450 enzyme profiles, and P450 enzyme polymorphisms.

For reactions to pass properly, esterases, such as paraoxonase, play a key role. One of the first studies conducted on rats by Chambers and Chambers [55] indicated that desulfuration of CPF in liver microsomes is 100 times greater than that in brain microsomes. The research by Choi et al. [49] showed that during the decomposition of CPF, the resulting products, such as 3,5,6-TCP, DETP, and DEP, in human hepatocytes are very similar to those in rodents.

### 3.3. Exposure Pathways of CPF in Humans

#### 3.3.1. Exposure to CPF in Humans by Oral Administration

The literature data indicate that in the era of such a high level of food contamination with chemicals of different origins, both adults and children are exposed to the consumption of higher doses of various pesticides, including, in particular, CPF [56,57]. However, according to Bradman et al. [56], children are exposed to higher doses of CPF compared to adults. This is related to their lifestyle: children eat more fruit and vegetables every day, and play on the floor and on the ground, putting different things in their mouths [56,58]. Moreover, children have largely functional immaturities of intestinal epithelial cells, which results in easier absorption of this compound [59]. According to Lehman-McKeeman [60], it cannot be ignored that if CPF is consumed as a residue in food or when other products are present in the gastrointestinal tract, its absorption may be less efficient, and its toxicity can be lower than when it is administered in its pure form into the empty tract. However, it is important to remember that lifestyle; the abuse of substances such as alcohol, nicotine, and drugs; and spicy foods can irritate the human digestive tract, allowing CPF to absorb more easily into the bloodstream, which distributes it throughout the organism. Therefore, there may be significant discrepancies in the results obtained by various researchers.

A research experiment using a single oral dose of 0.5, 1, or 2 mg/kg CPF in humans showed that the amount of recovered compound or its metabolite in urine and blood ranged from 20 to 35% [53]. As observed by Eaton et al. [46], the relative bioavailability of CPF may have been higher. It could have resulted from the fact that the percentage of the absorbed oral CPF dose in this study might have exceeded the estimated one based on the metabolites in urine or blood alone. Some amounts of the absorbed CPF could have been eliminated by other pathways, i.e., feces or breath. In turn, in other studies conducted by Mattsson et al. [61], the recovery of CPF in urine was 70% after a single oral dose. Such differences in the absorption of CPF after oral administration may suggest that the physical form and other properties of this preparation have a significant impact on its absorbability [46].

Therefore, for CPF, a health risk assessment was made on the basis of the acceptable daily intake (ADI) values which are 0.001 mg/kg body weight (b.w.)/day, throughout a lifetime. In contrast, the acute reference dose (ARfD) determined for these compounds is 0.005 mg/kg b.w./day. These amounts can be absorbed by the human body in no more than 24 h without risk to consumers’ health [62].

#### 3.3.2. Exposure to CPF in Humans by Inhalation

The most important occupational group exposed to CPF and other organophosphate pesticides through direct transdermal contact and by inhalation during the preparation of spraying solutions, loading of sprayer tanks, and application of pesticides are farmers.

Many studies have investigated human exposure to CPF by inhalation. Such research was undertaken because many insecticides used for pests in residential areas, such as termites, fleas, and lice, are based on CPF [62]. Its widespread use in houses exposed residents to this agent by inhalation. Therefore, based on a decision by US EPA [63], CPF was almost completely eliminated from sales to the public.

The literature data indicate that CPF is considered as a compound that easily penetrates the lungs after being sprayed in the air, although studies on humans and rats do not fully confirm this observation [64,65]. The study by Garabrant et al. [65] compared employees who had direct contact with CPF during the production process with people who were not exposed to it. There were no differences in the occurrence of central and peripheral nervous system symptoms in both groups. In addition, although the studies on rats showed that exposure to high doses of CPF in the air may cause inhibition of cholinesterase, the rate of absorption or the bioavailability of this compound from air is not fully understood. Therefore, it seems unlikely that concentrations of CPF in the air of less than 10 μg/m^3^ could cause inhibition of erythrocyte AChE activities or plasma butyrylcholinesterase (BuChE) in humans [46].

Regarding the in vitro studies of CPF in human cell lines representative of the respiratory system, evidence for the cytotoxic activity of pesticides is accessible. According to Oostingh et al. [66], CPF influences the viability of alveolar epithelial cells and T lymphocytes, significantly reducing it. In bronchial epithelial cells, the response was more variable: at low to medium concentrations of chlorpyrifos, an increase in the induction of IL-6 and proinflammatory cytokine tumor necrosis factor (TNFα) promoters was observed, especially in the presence of an additional stimulant, rhTNFα. These results indicate that in the presence of ongoing inflammation, which can be caused by, for example, even a common cold, additional exposure to organophosphate may result in increased disease intensity, which may increase the risk of developing chronic inflammatory diseases such as asthma and COPD [67].

#### 3.3.3. Exposure to CPF in Humans by Dermal Absorption

Adverse effects from the consumption of sub-toxic doses of CPF by both oral admission and inhalation have been proven in many studies. However, there is still a general perception that skin exposure to CPF is not as dangerous or as relevant as other routes of exposure. Therefore, especially in developing countries, both farmers and others involved in the production, transport, sale, and use of pesticides have not paid sufficient attention to skin exposure to CPF. This compound is absorbed through the skin, which may result in systemic poisoning. There is evidence that CPF and its metabolites accumulate in the skin, prolonging the exposure time and causing even more adverse long-term effects [68].

The efficiency of CPF absorption through the skin tends to be lower and the absorption rate is slower compared to other routes of exposure and depends on the solvent used. Even a single cutaneous application of CPF in ethanol for 4 h in humans causes an absorption of 4.3% of the applied dose, and the mean half-life is 41 h [69]. Exposure of pregnant Sprague-Dawley rats to a subclinical single cutaneous CPF dose of 30 mg/kg resulted in inhibition of AChE activity in the maternal and fetal brain within 24 h of exposure [70]. 

It should also be emphasized that the toxicity of chlorpyrifos is due to the fact that, unlike many other phosphorus-containing compounds, it is also a toxic chlorine-containing organic compound. The presence of chlorine brings with it all the problems associated with the possibility of its dehalogenation and the formation of decomposition products and secondary metabolites. Often, these compounds are more toxic than the parent compound. Chlorine-containing pesticides, including CPF, are capable of penetrating the placenta and exhibiting neurotoxic and immunosuppressive effects [71]. According to Bernardes et al. [71], although only about 10% of the applied pesticide dose is absorbed by the human body, its percutaneous penetration is increased due to the use of lipid solvents. This may result in an increased risk of intoxication, especially in people who are professionally involved in the production and application of pesticides.

The literature data even indicate a possible correlation of sub-toxic CPF doses with neurotoxic effects. According to Lim et al. [72], the application of CPF to the skin for 7 days to 3 weeks at a dose of 1/5 of the LD50 level was able to induce neurotoxic effects such as decreased serum cholinesterase activity, decreased hippocampal neuronal density, and increased expression of GFAP (glial fibrillary acidic protein). Low CPF doses of 1/10 LD50 applied to the skin resulted in decreased levels of neurotoxicity. In the initial phase of neurotoxicity, a glial reaction in the form of GFAP expression was observed as a result of the shorter application of a low CPF dose to the skin [72]. Taking into account the above studies and their results, skin exposure to CPF should be avoided.

The results obtained from field studies with applicators and residents have shown that exposure to CPF may occur as a result of its widespread use, both outdoors and indoors. Inevitably, professional workers are exposed unintentionally and unavoidably as evidenced by the presence of 3,5,6-TCP in urine, which was observed in workers dealing with the application of CPF [73].

Many research studies have been devoted to the effects of CPF on skin exposure in people working with this substance and children [64,74]. The research conducted by Atabila et al. [64] concerning household dust, in which the presence of CPF at a concentration of 230–710 ng/g was detected, showed that exposure to domestic dust did not have a significant impact on the health of residents. Eaton et al. [46] presented studies regarding the amount of CPF on surfaces in farmhouses and in places where it was recently used to control pests. CPF values were found to range from 10 to 210 ng/cm^2^. Based on these results, the scholars estimated the potential contribution of dermal CPF absorption to the total concentration of urinary 3,5,6-TCP. The obtained results indicated no correlation between the concentration of CPF in homes and the concentration of 3,5,6-TCP in urine, which suggests that it probably does not constitute a risk to health [73].

#### 3.3.4. Exposure to CPF in Children

Some evidence also suggests that chronic exposure to organophosphorus compounds can be particularly harmful in the prenatal period and disrupt the development of the child’s nervous system [55,75]. Women exposed to pesticides may have a disturbed hormonal balance, which may affect the reproductive system and fetus. Studies conducted in rats chronically fed with corn oil containing CPF at a dose of 6.75 mg/kg b.w. showed that the main places of accumulation of this agent in the organism are fat tissue, the liver, kidneys, and ovaries [76].

Studies were conducted in children exposed to different levels of CPF in the uterus (e.g., detected in umbilical cord blood, with amounts ranging from 3.17 to 360 pg/g in the 2nd to 3rd trimester). CPF easily crosses the placental barrier during pregnancy. The analyses showed that during the first 3 years of life, children showed delays in psychomotor and mental development [59]. Studies conducted by the US EPA [63] in pregnant rats exposed to different levels of CPF indicated that CPF in nursing rat pups influenced the height of the cerebellum, and thus resulted in damage to the architecture of the developing brain, negatively influencing cognitive functions. In the future, such damage may affect their whole life, causing impairments in, e.g., motor activity, learning, and memory. In addition, as reported by Eskenazi et al. [58], exposure of children and infants to CPF may cause lower IQ (intelligence quotient), developmental delay, ADHD (attention deficit hyperactivity disorder), and autism [77]. This was confirmed by the research studies conducted by UC Davis MIND Institute on pregnant women who lived near agricultural regions where CPF was used. It was found that in these women, there was an increased risk of having a child with autism. In addition, a study by Bölte et al. [78] and Hertz-Picciotto et al. [79] indicated that the risk of autism in children increased three-fold when women in the second trimester of pregnancy lived within a mile of agricultural regions treated with CPF.

### 3.4. Toxicity of CPF to Other Mammals 

#### 3.4.1. Acute Toxicity of CPF

**Figure 3:**
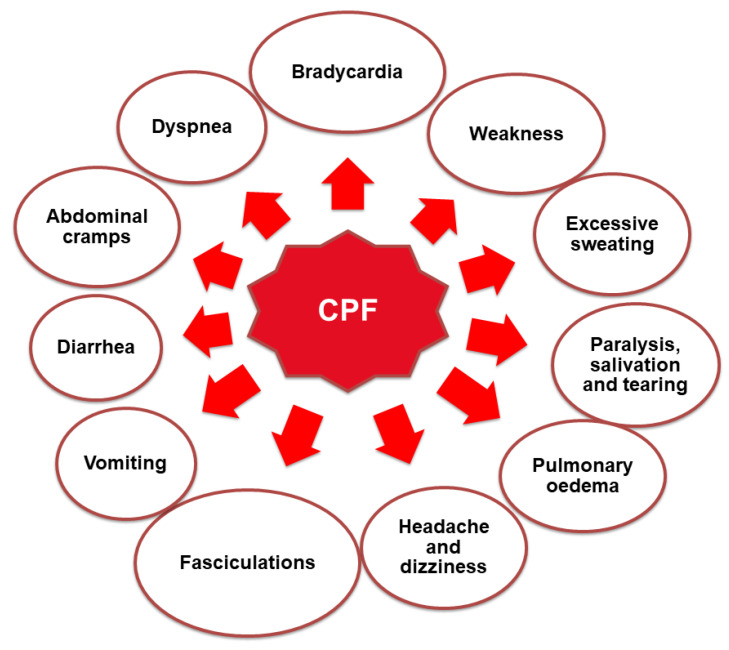
Undesirable health effects of exposure to CPF.

On the basis of acute systemic toxicity, it is possible to evaluate any undesirable effect on organisms occurring within 24 h after the application of a single or multiple doses of the test substance. This substance can enter the organism by inhalation of vapors or aerosols, ingestion of residues in the diet, and dermal exposure [46,80]. This may occur due to deliberate or accidental short-term exposure [81] and can cause many diseases (Figure 3). When acute systemic toxicity occurs, at the beginning, there might be symptoms such as headache or rash, convulsions or coma later, and in the last stage, it can even lead to death. Therefore, at the initial stage of research on a given substance, tests are mainly conducted on animals, such as mice, rabbits, and/or dogs, which allow for a prediction of how the substances may behave in humans [82].

During the Pesticides Peer Review expert meeting in April 2019, the toxicological profile of CPF was discussed on the basis of the guidelines SANCO/10597/2003-rev. 10.1, in which its absorption through the skin and the identification of endocrine-disrupting properties were assessed. Therefore, in order to determine its toxicological profile, one should take into account its acute toxicity and genotoxic potential, which ought to be assessed at appropriate levels. From a toxicological point of view, both the parent compound and metabolites may pose a risk for plants, animals, and humans [83].

Regulatory authorities such as the EFSA and US EPA verify the toxicity tests provided by producers of pesticides [84]. Toxicological risk assessments are performed using animal tests to allow for more accurate evaluation and identification of the adverse toxic effects of tested compounds and their performance is commissioned by external commercial laboratories whose test reports are not published [85]. 

The only reliable studies come from academic and industry-sponsored toxicity research, which is not always consistent and can lead to fundamentally different conclusions regarding pesticide safety. For example, on the basis of independent in vitro and in vivo epidemiological studies, the results of the adverse effects of exposure to CPF on health are presented. Thus, a negative influence on the development of the nervous system associated with lowered IQ in schoolchildren was found at exposure levels far below those recognized to affect brain development according to the industry-funded developmental neurotoxicity (DNT) studies commissioned for regulatory purposes [84,86].

#### 3.4.2. In Vitro and In Vivo Analysis of CPF Toxicity

Due to ethical reasons, in addition to animal models, for toxicity assessment of pesticides, other models that do not require the use of animals are used. For example, in silico and in vitro methods can be applied [87] before research is conducted on humans [88]. The final results of such tests allow for determination of the effects of exposure to large amounts of CPF and evaluation of the level of mortality. Moreover, according to Srivastava and Kesavachandran [83], depending on animal models, acute toxicity (LD50) can have different values: for example, for oral LD50 in rabbits, 1000 mg/kg; guinea pigs, 500–504 mg/kg; sheep, 80 mg/kg; mice, 60 mg/kg; chicken, 32 mg/kg; and for dermal LD50 in rabbits, 1000–2000 mg/kg.

Recent research studies have shown that in rats, an oral dose of CPF was fully absorbed, widely distributed, and extensively metabolized by hydrolysis and oxidation in the animal body, and it was mainly excreted in urine within 48 h [62]. According to EFSA [83], the acute oral toxicity for rats was 66–195 mg/kg b.w. while in 2019, EFSA reported an acute oral toxicity for rats of 66–223 mg/kg b.w. [62]. In turn, as reported by EFSA [62], acute toxicity measures for CPF in rats by dermal and inhalation were, respectively, 1250–2000 mg/kg b.w. and > 1.0 (mg/L) air per 4 h (whole body).

In vitro studies showed that the liver microsomes of mouse, rat, and human more readily produced a detoxication product called 3,5,6-TCP than an activation product called CPF-ox. Thus, based on this study, it was found that the formation of CPF-ox was three times lower than the formation of 3,5,6-TCP. In the available acute toxicity research, EFSA experts determined the toxico-kinetic values for CPF (i.e., area under the blood concentration/time curve, concentration achieved at peak blood level, time until peak blood levels are achieved, and half-life), with high, moderate, and low acute toxicity when administered via respiratory, dermal, and oral routes, meeting the criteria for classification as toxic if swallowed (Acute Tox. 3, H301) and harmful in contact with skin (Acute Tox. 4, H312), according to the CPF criteria. Interestingly, based on Regulation (EC) No 1272/2008 Annex IV regarding human health, CPF did not cause potential eye and/or skin irritation, sensitization, and/or phototoxicity [62].

Table 1 presents selected reports of studies on exposure to CPF and its effect on various animals. Research on chickens by Begum et al. [89] showed that after the oral administration of CPF in a high single dose of 36 mg/kg b.w., after 2 h, excessive salivation and agitation followed by drowsiness combined with greenish bloody diarrhea was observed. Further, chickens were unable to stand and sit on joints with their folded toes and finally, within 4–36 h of dosing, all treated birds died [89,90,91]. In turn, a study on old northern bobwhite was carried out in which the administration of CPF at a dose of 21.6 mg/kg b.w. resulted in a ruffled appearance and lethargy and the body weight of both sexes of the birds was significantly decreased [90]. For example, studies conducted on wild birds fed with CPF did not provide conclusive results on the impact of this pesticide and the above signs were not observed.

In addition, studies were conducted on male rats that were orally administered CPF at a dose of 2.7, 5.4, and 12.8 mg/kg for 90 days to assess the toxic changes that occur in testicular histology, testosterone concentration, sperm dynamics, and testicu-lar marker enzyme activities. Chronic exposure of male rats affected folli-cle-stimulating hormone and caused a decrease in the concentration of testosterone and thus a reduction in the sperm count and motility [103]. Another study, in which rats were treated orally (via gavage) with a dose of 0, 0.5, 2.5, or 15 mg/kg Pyrinex Technical on days 6–15 of pregnancy, revealed that the highest dose led to low changes in food consumption, tremors in 3/21 rats, and body weight gain. From these data, the NOAEL for the maternal function assessed for Pyridex reached 2.5 mg/kg [46].

It was noted that the only the long- and short-term effect of long-term CPF con-sumption inhibited AChE activity while high doses led to excessive endogenous cho-linergic stimulation, causing typical cholinergic symptoms. In contrast, animal studies showed that exposure to CPF even at low doses but at key developmental times can cause permanent changes in brain functions [104]. Grabovska and Salyha [105] stated that exposure to CPF in female rats leads to neurobehavioral impairments in the off-spring. According to EFSA, for long- and short-term exposure to CPF at 0.1 mg/kg b.w./day, no adverse effect was observed. This level of CPF was found on the basis of a significant decrease in AChE activity in red blood cells at 1 mg/kg b.w./day in a 90-day and 2-year study on rats and dogs. In addition, there was no potential for CPF car-cinogenicity when administered to mice or rats and no data on its immunotoxic poten-tial [62].

#### 3.4.3. Genotoxicity of CPF

The Renewal Assessment Report (RAR) contains an assessment of toxicity in in vitro and in vivo regulatory studies. It also discusses the effects of CPF on gene mutation, chromosome aberration, unscheduled DNA synthesis, and in vitro studies in somatic cells [62]. Research on animal cell lines showed that CPF does not induce gene mutations in vitro. In addition, CPF was also found to be unable to induce chromosome aberration in in vitro tests. In turn, after the application of CPF, Abdelaziz et al. [106] noted chromosomal aberrations in vivo and DNA damage in Comet assays both in vitro and in vivo [107,108]. Cui et al. [109] observed the effect of CPF on unscheduled DNA synthesis. However, in vivo somatic cell tests of mouse bone marrow micronucleus in RAR provided negative findings for CPF. The above results require additional research, including a new Comet test [110], to confirm the results observed in in vitro tests and one cannot ignore the fact that CPF may have genotoxic potential. Regarding CPF, there are also no unambiguous reports on its genotoxic potential; however, it cannot be excluded that CPF has DNA-damaging potential despite the lack of such reports [111].

According to EFSA [112], CPF elicits oxidative stress in various tissue and cell types, which results in damage to all vital macromolecules, including proteins, lipids, and DNA. Thus, oxidative DNA damage can be followed by DNA single- and double-strand breaks, which may also interact with biological molecules to disrupt normal DNA synthesis and repair, influencing the mRNA expression profiles in brain cells [107]. As suggested by Ojha et al. [113], 24 h post-treatment, acute and chronic exposure to CPF caused significantly marked DNA damage in rat tissues, namely the brain, liver, spleen, and kidney. In turn, in a study using fetal liver hematopoietic stem cells, Lu et al. [114] observed that CPF can also cause DNA damage through topoisomerase II inhibition. The epidemiological studies conducted by Hernández and Menendez [115] noted an important association between CPF exposure and infant leukemia. Thus, exposure to CPF in dam rats may lead to neurobehavioral impairments in offspring.

The results obtained by Serpa et al. [116] confirm the genotoxic potential of CPF. They demonstrated the genotoxic effect of CPF in vitro in human leukocytes at a concentration of 35 μg/mL by causing the following alterations: micronuclei, numerical chromosomal abnormalities, and apoptotic cells [117].

#### 3.4.4. Endocrine-Disrupting Properties and Developmental/Reproductive Toxicity of CPF

Numerous pesticides are recognized for their endocrine-disrupting properties. It is worth noting that there is literature data showing the pleiotropic effects of endocrine-disrupting substances, including pesticides, on human health. Both single chemical substances and mixtures of chemical compounds can cause neurological and immunological toxicity and carcinogenesis in animals and humans. Exposure to pesticides is highly harmful to offspring following in utero exposure and may lead to birth defects and effects on growth and normal development [117,118]. Due to their high activity, ease of penetration into living organisms, resistance to environmental degradation, and influence on germline, pesticides, as endocrine disruptors, have been associated with intergenerational epigenetic inheritance. Increasingly more evidence is emerging to suggest that epigenetic information, which is faithfully passed on between cells during mitotic division, can also be passed on between generations. Therefore, it is concluded that the health phenotypes of the offspring can be acquired in an epigenetically inherited manner as a result of the environmental exposure of the parents. This environmental risk of disease has been shown to be transmitted to offspring through epigenetic mechanisms through both female and male germlines [119]. While most of the evidence for this mode of inheritance of disease comes from maternal exposure during pregnancy, it has also been shown that pre-conception paternal exposures are also important in determining disease outcomes in offspring [120,121]. Organophosphates, including CPF, are a toxic class of insecticides that are able to influence the phosphorylation of various proteins, and their toxicity is related to inhibition of the enzyme acetylcholinesterase. Exposure to organophosphate pesticides during pregnancy results in their transfer to the fetus via the placenta or amniotic fluid, which in turn significantly affects the child’s development [122]. Exposure to organophosphates causes, in addition to congenital defects, cognitive and neurobehavioral deficits. These pesticides increased the risk of breast cancer, and maternal exposure was also associated with childhood development of acute lymphoblastic leukemia. Maternal genotoxic exposition can induce non-homologous chromosome rearrangements and initiate neoplasm [123,124].

The chronic toxicity of CPF is difficult to determine, and in order to obtain more information, selected animal species should be exposed to regularly repeated low doses of this pesticide [125]. Defects in newborn development, rare/unusual tumors, endocrine disruption, or nerve disorders are some of the typical chronic effects that occur after contact with the pesticide. Therefore, it is easier to evaluate the acute toxicity of a pesticide than determine the dose of its chronic toxicity [82].

Recent studies have suggested that exposure to CPF can also affect the endocrine system, in particular thyroid and adrenal gland homeostasis, which was observed in animal and human models [125,126]. Numerous in vitro studies conducted by Aldridge et al. [98] indicated that CPF may operate by other mechanisms. In addition, they showed that exposure to this substance below the toxicity threshold may have a destructive effect on neural cell development, cell differentiation, and synaptogenesis. In vivo mouse model studies conducted by Salazar-Arredondo et al. [127] indicated serious defects in spermatogenesis, resulting in a reduction in sperm quality. Therefore, CPF could be considered as an endocrine-disrupting compound [128]. A study on the toxicity of CPF in two generations of rats attested that only the highest dose of 5 and 10 mg/kg b.w./day affected the reproductive performance and reduced pup growth and viability at NOAEL of 1 and 3 mg/kg b.w./day [111]. 

Studies on the effects of CPF on developmental toxicity in animals such as rats, rabbits, and mice indicate that rats are the most sensitive animals. At the highest doses of CPF, inhibition of AChE activity in erythrocytes regarding maternal toxicity increased post-implantation loss in rats while in rabbit, decreased fetal size and increased post-implantation loss occurred, whereas in mice, no developmental toxicity potential was determined. In turn, no negative developmental effects were observed in rabbits and rats after exposure to CPF. On this basis, it was concluded that CPF does not function as an endocrine disruptor in humans [129]. All experts agree that there is no need for an assessment of endocrine disruptors in humans after exposure to CPF. Such a decision was made because in all the analyzed studies in animals, NOAEL (no observed adverse effect level), LOAEL (lowest observable adverse effect level), and MTD (maximum tolerated dose) for this pesticide were established on the basis of inhibition of AChE activity in erythrocytes and clinical symptoms were observed only at the high doses of this compound [62,111].

The epidemiological studies conducted by Tian et al. [130] and Marasinghe et al. [131] showed that human exposure to CPF reduced the head circumference of infants, decreased birth weight, and increased the risk of prostate and lung cancer. According to Peiris and Dhanushka [132], in vivo animal model studies indicate that CPF causes certain defects in spermatogenesis, resulting in reduced semen quality, whereas Mandal and Das [133] added that CPF interferes with the endocrine functions of the pituitary and hypothalamus in rats and affects spermatogenesis, which may also be reflected in people working with this pesticide. Pallotta et al. [134] conducted in situ hybridization with X and Y sex chromosome probes on sperm samples exposed to CPF concentrations (1, 5, 10, 25, and 50 μg/mL), observing that a significant proportion of sperm had peculiar morphological malformation. The authors reported that at CPF concentrations of 10, 25, and 50 μg/mL, this compound can cause genotoxic effects in spermatozoa, impairing their ability to fertilize. Heikal et al. [135] showed that the decrease in sperm motility was caused by a reduced ATP content while the mitochondrial activity and impairment of microtubular sperm structures were important for normal sperm physiology. 

The research conducted on rats by Li et al. [94] attested that CPF may cause inflammation and hormonal changes and have effects on the brain–gut axis and gut microbiota. Moreover, this substance can regulate communication between the brain and the gut by pathways such as the production of gut hormones, stimulation of the hypothalamic–pituitary–testis axis to release hormones, and acceleration of systemic inflammation. Furthermore, Mittal et al. [136] indicated that during development, the central nervous system is particularly susceptible to CPF. As a neurotransmitter functioning between the gut and the central nervous system, CPF plays a major role in maintaining gut homeostasis, including absorption, the gut microbiota, the immune system, and motility.

Obesity is not only simply excessive fat accumulation; it is also associated with low-grade chronic inflammation, which is the main factor inducing insulin resistance [137]. This was confirmed by the research studies conducted by Peris-Sampedro et al. [138] on farm animals exposed to CPF. They showed that these animals may develop hyperinsulinemia and hyperlipidemia, so obesity can develop. In addition, in vitro and in vivo research conducted by Condette et al. [139] indicated the possibility of disorders of the intestinal epithelial cell zonula occludens-1, which increases intestinal permeability and plasma lipopolysaccharide levels [140,141]. Liang et al. [100] observed that CPF-fed mice had a higher fasting glucose and insulin concentration compared to the respective control groups, suggesting that CPF may induce insulin resistance and disrupt glucose homeostasis, thereby causing obesity and increasing the risk of developing chronic diseases, e.g., type 2 diabetes.

Literature data indicate that CPF may act as a weak estrogenic compound, affecting the expression of estrogen receptors (ERs), and demonstrating antiandrogenic, thyroid, and aryl hydrocarbon receptor (AhR) agonist activity [142,143]. In vitro results indicate that CPF stimulates cell proliferation in the human breast cell lines MCF-7 and MDA-MB-231. In ER-responsive cells, it most likely acts through a mechanism involving ERα activation. at a dose of 0.01 mg/kg b.w./day, CPF administered to adult female rats increased the number of mammary ducts and stimulated cell proliferation and PgR expression. However, at the same time, at a higher concentration (1 mg/kg b.w./day), this compound decreased serum estradiol and progesterone levels. A change in mammary gland morphology (including duct thickness and branching) was observed after exposure to CPF (0.1 and 2.5 mg/kg/day) in adult female rats. Breast tubulogenesis induction by activation of the AhR pathway was observed in the CPF-exposed MCF-7 line [128,144,145].

#### 3.4.5. CPF Developmental Neurotoxicity

CPF present in the environment may be directly or indirectly developmentally neurotoxic. The effects of developmental neurotoxicity will depend on the stage of brain development, the dose used, and the duration of exposure [146]. Available animal studies indicate that sub-toxic exposure to CPF has a detrimental effect on the behavior and development of the central nervous system. During a recent meeting of experts in April 2019, studies on the effect of CPF on developmental neurotoxicity were discussed [62,111]. Undeniably, research on mice and rats conducted in recent years has allowed for a better understanding of the developing brain’s sensitivity to the neurotoxic effects of CPF after the consumption of contaminated food [74]. The developmental neurotoxicity was studied in pregnant rats exposed to various levels of CPF at 0.3, 1, and 5 mg/kg b.w./day from day 6 of gestation to PND 11. The obtained results showed that the applied doses significantly lowered the number of individuals displaying neuropathology. CPF also affected the juveniles’ behavioral ontogeny: they had difficulty in learning and memorizing. However, other studies indicate that sub-toxic exposure to CPF in developing organisms and the assessment of neurotoxicity [62] should be further investigated as the available studies appear to be controversial and do not fully clarify this issue [147].

One of the causes of neurodegeneration is the effect not only on the peripheral nervous system but also on the central one, in which not only cholinesterases but also decarboxylases are extremely important, catalyzing the formation of the necessary hormones to control many processes. Recently published assessments of the effects of CPF on these enzymes have shown a decrease in activity, which leads to the development of autism, Parkinson’s disease, etc. In addition, the penetration of CPF is shown not only in the placenta, liver, and kidneys but also in the brain.

CPF and its derivatives (mainly CPF-ox) are potent irreversible inhibitors of AChE, which is responsible for the breakdown of the neurotransmitter acetylcholine. The CPF oxygen analogue—CPF-ox—is formed in the process of catalysis by the cytochrome P450-dependent mono-oxygenase system. The decreased activity of AChE after exposure to CPF leads to increased activity of cholinergic synapses in neurons and neuromuscular junctions. The exposure of mammals to high concentrations of CPF and its derivatives can cause acute lethality from respiratory failure, resulting from both central and peripheral effects [148]. The action of CPF may disrupt the spatial structure of the enzyme by attacking the active hydroxyl group of the AChE serine. In this way, CPF and its derivatives inactivate the enzyme and hinder the hydrolysis of the hydroxyl group. AChE inhibition is therefore irreversible and leads to the accumulation of the neurotransmitter acetylcholine and ultimately neurotoxicity [149]. 

As noted in the studies by Rauh et al. [77], frontal and parietal cortical thinning was observed in children exposed to CPF at a prenatal age. Moreover, these results coincided with regulatory studies, in which similar structural changes were determined in the developing brain of rats following exposure to CPF. Moreover, a CPF dose-related decrease in plasma cholinesterase (ChE) and erythrocyte AChE activities was observed [46]. In turn, AChE activity in the brain was decreased after the application of 1 and 5 mg/kg b.w./day in the treated groups. On the basis of these results, a rat maternal LOAEL was proposed at 0.3 mg/kg b.w./day (based on the decrease in ChE and AChE activities) while a rat pup NOAEL was calculated at 1 mg/kg b.w./day (based on the decrease in the viability index, food consumption, body weight, body weight gain, and absolute brain weight and an increase in the relative brain weight in organisms exposed to a dose of 5 mg/kg b.w./day) [77]. 

### 3.5. Occurrence of CPF in Food and Risk Assessment

CPF’s initial form/parental compound or its metabolites have the ability to remain in the soil, water, and atmosphere, and penetrate many agri-food products, which may pose a danger to living organisms [150]. In products of animal origin, it is present mainly as a result of the consumption of contaminated feed by animals and on fruit and vegetables due to the spraying of farm areas during the growing season [151]. 

For pesticides, maximum residue levels (MRLs) are established for food and drinking water to ensure the health of living organisms. As observed in the study by Jankowska et al. [152], CPF residues in drinking water and food often occur at relatively low detected values, which makes dietary risk assessment difficult. Therefore, it was available for a long period of time before decisions were made to withdraw it from the market because of its toxicity. Solomon et al. [153] indicated that pesticides such as CPF tend to accumulate in food products, and their universality regarding uses on various agricultural crops contributes to a significant share in the human diet. In addition, the rate of absorption by organisms after ingestion with food and water poses a toxicological threat [152,153]. The studies conducted by Yuan et al. [154] and Slotkin [155] show that CPF is one of the most frequently detected pesticides in food, with the highest rate of 38.3%.

It is well known that food quality is closely related to the place of origin and environmental pollution. Even low concentrations of pesticides, i.e., below the acceptable residue limit (MRL), in food can cause negative health effects, especially when the exposure is prolonged over time [156]. On the basis of their research, Riederer et al. [157] estimated the daily intake of CPF, proving that an individual consumes approximately 2.1 × 10^−4^ mg/kg b.w. of this substance in various products during each day.

EFSA [104] reported that the highest chronic exposure calculated for Irish adults was 61.2% ADI while the highest acute exposure calculated for apples was 54.9% ARfD. Studies on pesticide monitoring of apples from South Kazakhstan showed that CPF exceeded safety ARfD: 136% for adults, whereas 742% for infants [158]. As indicated by Mojsak et al. [159], on the basis of available toxicological results, EFSA [83] decided to lower the reference values for ADI and ARfD to ensure that the doses absorbed by the human body are low enough to not affect human health.

Based on monitoring by EFSA in the EU territory in 2020, several non-approved EU pesticides, including CPF, were found in randomly sampled foods such as carrots, pears, potatoes, and rye grain at levels above the permissible limits [160]. Studies on pesticide monitoring in vegetables and fruit in Valencia in 2007–2011 demonstrated the constant presence of organophosphorus compounds [161], with CPF concentrations of 0.02 mg/kg in carrots and potatoes and 0.04 mg/kg in cucumbers and tomatoes. CPF residues (0.01 mg/kg on average) were also recorded in cauliflowers from crops sprayed with a preparation containing this compound as the active substance [162]. However, for fruit, the content of CPF was 0.01–0.47 mg/kg in apples and 0.02–1.96 mg/kg in bananas [158,162], with no MRLs being exceeded [163]. 

**Figure 4:**
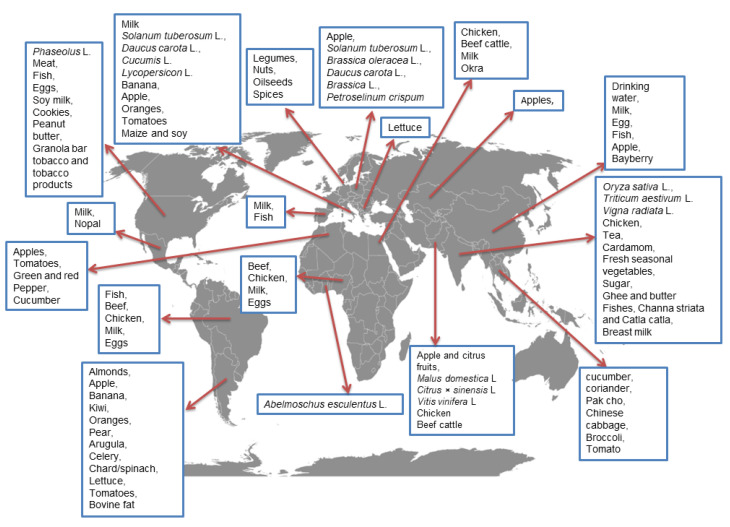
Occurrence of CPF residues in food in various parts of the world [37,158,166,167,168].

CPF residues were also reported in agricultural products originating from south-eastern Poland. Their presence was found in the following samples: 6.6% of potatoes (0.01–0.07 mg/kg), 31% of broccoli (0.02–0.07 mg/kg), 56.1% of carrots (0.01–0.08 mg/kg), 57.1% of cabbage (0.01–0.03 mg/kg), and 100% of parsley (1.45 mg/kg) (Figure 4) [164]. Moreover, studies on CPF in Polish fruits and vegetables over the course of a long period of research, i.e., 2007–2016, were conducted by Mojsak et al. [159]. They observed that among 3530 samples, CPF was present in 10.2% and the highest levels were found in broccoli at 1.514 mg/kg. In addition, this study re-calculated the short-term risk assessment for apples, broccoli, and carrots based on new values for ARfD (0.001 mg/kg). It was shown that children and infants had a greater dietary risk due to their higher tendency to consume fruit and vegetables on a per body weight basis. The long-term exposure values calculated for adults and children do not constitute a health risk. MRL exceedance was identified in a greater number of samples according to the new established values. The study conducted by Essumang et al. [165] discussed the presence of CPF in edible musk from Ghana of approximately 1.32 mg/kg despite the non-use of pesticides during cultivation.

Pesticide monitoring in India also indicated the presence of CPF in rice and wheat, not only in plants grown on farms that used agrochemical treatments but also in organic ones, although in trace amounts. The reason for this is the mass use of these preparations in agriculture [166]. For example, Riederer et al. [157] noted a decrease in the content of CPF in subsequent links in the food chain, from 0.194 mg/kg in beans, nuts, and legumes to 0.0024 mg/kg in meat, fish, and eggs (Figure 4).

CPF residues were also found in chicken and cattle meat, both from a local Egypt market and imported from India and Sudan [169], and the contents of CPF ranged from 0.015 to 0.012 mg/kg. Moreover, the highest CPF concentrations in samples of beef and chicken from Brazil were, 17.5 and 0.08 μg/ kg, respectively [170]. Due to its nutritional value, milk and milk products have a tendency to accumulate harmful chemical compounds. CPF residues were detected in milk in Italy, Brazil, and Mexico (0.005, 0.003, and 0.020 mg/kg, respectively) [171]. In addition, in all milk samples tested, the detection frequency of CPF was 76% and the highest concentration level was 45.7 μg/L [170]. In studies conducted by Hartle et al. [172], it was found that the concentration of CPF in breast milk ranged from 4.2 to 54.6 pg/g. Moreover, this milk was pasteurized and marked CPF ranged from 3.5 to 34.4 pg/g, which confirms the durability and maintenance of CPF at high values despite being subjected to preservation processes.

CPF is present not only in vegetables and fruits and in flour (soy and wheat) but also in their processed products: in flour products, canned food (in Bulgaria, it has long been shown that after processing tomatoes into ketchup, pesticides are preserved), honey, and various nuts (even in sweet products with nuts) that are considered beneficial for health, as they contain “good” fatty acids in lipids, which hold CPF.

In turn, the research study conducted by Oliveira et al. [173] proved the presence of CPF of approximately 36.1% compared to all labeled insecticides in fish caught in the São Francisco River in Brazil. The content of CPF was detected both in the guts and muscles. In addition, it was found that farmed fish had higher detection rates (23%) for CPF than wild fish from the ocean (5%). This was related to the feed given to the farmed fish [174].

It cannot be overlooked that CPF residue exposure assessment has traditionally been carried out on a single product basis. However, a person may come into contact with the same substance every day several times not only through the diet but also drugs, such as tobacco and tobacco products, and through clothes made of cotton that has been treated with CPF and recommended for use by children and athletes [161]. The cumulative effect is particularly dangerous because it can lead to an increase in the toxic effect in a given person. Therefore, the chronic cumulative exposure should receive special attention to determine actual health risks.

## 4. Conclusions

CPF is degraded in the environment by both biotic and abiotic methods, including physical and biochemical methods [16]. Therefore, the development of effective and economical approaches to decontamination and detoxification of CPF-contaminated environment is required. Researchers have studied various physicochemical methods for CPF remediation in contaminated environments such as incineration, dumping in deep oceans, burning in open pits, and advanced oxidation processes. However, these processes lead to the generation of secondary pollutants that exhibit higher toxicity and the ability to accumulate recalcitrant residues, which requires further treatment and is costly, chronophagous, unecological, and technically challenging [168,175]. Therefore, the utilization of indigenous microorganisms for CPF removal from environmental matrices has become the focus of researchers because of its efficient, economical, and eco-friendly nature [168]. 

The negative effects caused by CPF in the environment are mainly confined to changes in the soil microflora population, which may result in, among others, inhibition of nitrogen fixation, changes in the activity of soil enzymes such as phosphatase and beta-glucosidase, and changes in the migration of antibiotic resistance genes. On the other hand, in the aquatic environment, genotoxic and neurotoxic effects have been observed, and an increased level of oxidative stress in freshwater organisms has also been detected. Therefore, it seems crucial and justified to introduce modern bioremediation methods that include selected microorganisms, including endophytes, and use nano-materials that support processes that reduce the risk of CPF in the environment.

CPF has been detected in biota at various trophic levels due to the fact that it can be transmitted by air currents over long distances (transboundary transport). This can lead to adverse effects on human health and cause serious environmental effects, justifying the monitoring of CPF in various environmental compartments.

Regarding human health, the available epidemiological studies present irrefutable evidence of the harmful effects of CPF on human health. Moreover, a serious problem seems to be the discrepancies in the reports that discuss the toxic effects of CPF on human health. In many works, it was observed that the toxicity of these compounds resulted, to a great extent, from the high doses used, which significantly affected the genotoxic potential and endocrine system and/or inhibited AChE activity in erythrocytes. Although CPF has been withdrawn from the EU market, this does not mean that humans will not be exposed to it in the future. Therefore, continuous monitoring of the residues of CPF in food and the environment is important in order to prevent this compound from ever reaching EU markets with imported products. Furthermore, it is necessary to continue research to determine the changes occurring at the cellular and tissue level in humans and animals at all stages of life and to investigate how it will affect the body several years after exposure to CPF.

## Figures and Tables

**Table 1:** Reports of the studies of mammalian exposure to CPF.

Species	Dosage/Route/Type	Effect	Ref.
Rats	Initial dose of 60 mg/kg, followed every 2 months with a single dose of 45 mg/kg	Deficits in learning	[90]
Rat pups on PND 1–6	Diet at a daily dose1234567of 1.5 or 3.0 mg/kg (by gavage in corn oil)	Decreased levels of mRNA for nerve growth factor, muscarinic M1, and reelin receptors, and an increase in glial fibrillary acidic protein mRNA and inhibited brain AChE activity	[92]
Rat pups PND 11–16	Daily dose 0.5, 0.75, and 1.0 mg/kg b.w.	For brain AChE inhibition being 1.0 mg/kg b.w./day	[93]
3-month-old Long-Evans rats	Diet at a daily dose of 0, 1, or 5 mg/kg for 1 year	No effects on learning or memory	[90]
3-week-old male Wistar rats	Daily dose 0.30 mg/kg b. w. normal fat	Significant increase in various hormones such as pancreatic polypeptide, gastric inhibitory polypeptide and monocyte chemoattractant protein 1 and tumor necrosis factor α	[94]
	Daily dose 0.30 mg/kg b.w. high fat	Significant influence on the gut microbiome and increased glucagon-like peptide-1	
Pregnant rats from GD 6 to PND 10	Daily dose at 0.3, 1, and 5 mg/kg b.w.	5 mg/kg: led to a decrease in pup weights and viability index (%) and presented cholinergic signs (fasciculatins, ataxia, tremors, etc.)12345670.3 and 1 mg/kg: 8–11% decrease in the cerebellum height to brain weight ratio	[95]
Pregnant rats from GD 14–20	10 mg/kg CPF (oral)	Reduced body mass gain in mothers during treatment and increased body weight gain in male offspring from PND42	[96]
Adult Wistar rats weighing 150–200 g	10 mg/kg b.w. 28-day oral exposure	decreased GSH-Px activity in blood	[97]
Male Sprague-Dawley adult rats	Doses of 0.1, 1, and 10 mg/kg b.w. once daily for 7 days (sunflower oil)	Inhibition of AChE activity by approximately 20%	[98]
CD-1 mice from GD 15–18	3 or 6 mg/kg/day (peanut oil)	3 mg/kg: approximately 10% brain AChE inhibition12345676 mg/kg: 40% brain AChE inhibition 24 h after last dose	[99]
Pregnant CD-1 mice from GD 14–17	6 mg/kg CPF (oral)	Concentration of 3,5,6-TCP found in the brains of fetuses was 250 ng/g and revealed decreased cognition in males and females	[100]
Pregnant guinea pigs starting approximately GD 53–55	25 mg/kg/day formulated in peanut oil, 10th day	Decrease in AChE activity in red blood cells by approximately 75%	[75]
3-week-old male C57Bl/6 and CD-1 (ICR) mice	diet at daily doses of 5 mg/kg (dissolved in corn oil) for 12 weeks	Can disturb glucose homeostasis and induce insulin resistance and effects on intestinal inflammation	[101]
Neonatal mice PND 10	a single oral dose (0.1, 1.0 or 5 mg/kg b.w.)	Induced effects are not related to the classical mechanism of acute cholinergic hyperstimulation, as the AChE inhibition level (8–12%) remained below the threshold required to cause systemic toxicity	[102]

AChE—acetylcholinesterase; GD—gestational days; PND—postnatal day; 3,5,6-TCP—urinary biomarker 3,5,6-trichloro-2-pyridinol; GSH-Px—glutathione peroxidase; body weight—b.w.

## Data Availability

Not applicable.
